# Co-implanting orthotopic tissue creates stroma microenvironment enhancing growth and angiogenesis of multiple tumors

**DOI:** 10.12688/f1000research.2-129.v2

**Published:** 2013-08-29

**Authors:** Per Borgstrom, Phil Oh, Malgorzata Czarny, Brian Racine, Jan E Schnitzer

**Affiliations:** 1 Sidney Kimmel Cancer Center, 10905 Road to the Cure, San Diego, CA, 92121, USA; 2 Proteogenomics Research Institute for Systems Medicine, 11107 Roselle St, San Diego, CA, 92121, USA

## Abstract

Tumor models are needed to study cancer. Noninvasive imaging of tumors under native conditions *in vivo* is critical but challenging. Intravital microscopy (IVM) of subcutaneous tumors provides dynamic, continuous, long-term imaging at high resolution. Although popular, subcutaneous tumor models are often criticized for being ectopic and lacking orthotopic tissue microenvironments critical for proper development. Similar IVM of orthotopic and especially spontaneous tumors is seldom possible. Here, we generate and characterize tumor models in mice for breast, lung, prostate and ovarian cancer by co-engrafting tumor spheroids with orthotopic tissue in dorsal skin window chambers for IVM. We use tumor cells and tissue, both genetically engineered to express distinct fluorescent proteins, in order to distinguish neoplastic cells from engrafted tissue. IVM of this new, two-colored model reveals classic tumor morphology with red tumor cell nests surrounded by green stromal elements. The co-implanted tissue forms the supportive stroma and vasculature of these tumors. Tumor growth and angiogenesis are more robust when tumor cells are co-implanted with orthotopic tissue versus other tissues, or in the skin alone. The orthotopic tissue promotes tumor cell mitosis over apoptosis. With time, tumor cells can adapt to new environments and ultimately even grow better in the non-orthotopic tissue over the original orthotopic tissue. These models offer a significant advance by recreating an orthotopic microenvironment in an ectopic location that is still easy to image by IVM. These “ectopic-orthotopic” models provide an exceptional way to study tumor and stroma cells in cancer, and directly show the critical importance of microenvironment in the development of multiple tumors.

## Introduction

Relevant animal models are vital to understand the processes involved in tumor progression and to develop new therapies ^1– 4^. Solid tumors can be followed in their native tissue when tumor cells are administered properly into their orthotopic tissue, or when tumors are chemically induced or arise spontaneously, for instance in genetically engineered mouse models. Though *in vivo* imaging systems are advancing rapidly, imaging orthotopic tumors within the animal, especially at the cellular level for extended periods of time dynamically and continuously, remains a significant challenge. Subcutaneous models that implant rodent or human tumor cells directly into the skin of animals have quickly become the most commonly used tumor models ^5– 7^, in part because these tumors are convenient, easy to implant, and are readily imaged outside the body ^4, 8^.

Recent advances in intravital microscopy (IVM) have made subcutaneous tumors even easier to image dynamically at higher resolutions in live rodents through dorsal skinfold window chambers ^7, 9, 10^. Standard light and fluorescence microscopy in this system can distinguish individual cells in the tumor so that many cellular events, such as cell migration, mitosis, pyknosis, apoptosis, and the growth of blood vessels, can be readily quantified. Intravital microscopy can also be particularly powerful for evaluating tumor imaging probes and therapeutic agents by visualizing at high resolution and quantifying tumor targeting, delivery, processing and efficacy *in vivo*, dynamically and continuously.

Tumor cell interactions with the surrounding tissue stromal environment, including extracellular matrix, local enzymes and proteases, vasculature, inflammatory cells, growth factors and hormones, can significantly affect tumor development ^11– 13^ and are, to a large extent, extensively altered or even missing when tumors are grown in ectopic environments such as skin ^14– 18^. Most orthotopic tumor models and especially spontaneous tumors are not readily amenable to IVM except possibly acutely for very short periods after surgical exposure, which frequently can be quite invasive. Moreover, injecting tumor cells properly to maintain an orthotopic tissue microenvironment can be quite difficult, in part because the orthotopic organ to be injected can be so very tiny in the mouse. Making sure that all of the injected cells enter and stay inside tiny organs can be quite challenging. Microsurgical techniques with stereomicroscopic imaging can help but greatly increase the labor per mouse.

Recently, we have successfully engrafted donor tissue from healthy rat organs and mouse prostate tissue with hormonally sensitive prostate tumor cells into the dorsal skinfold of mice carrying a window chamber for dynamic and continuous IVM imaging *in vivo* ^19, 20^. The implanted tissue maintained both tissue- and species-specificity, even expressing key organ-specific biomarkers ^19^. Here, we expand this tissue transplantation and revascularization model to multiple cancers by engrafting different donor tissues with various tumor spheroids to create novel ectopic-orthotopic (EO) tumor models that permit dynamic imaging by IVM while attempting to provide and maintain an orthotopic stroma microenvironment for the tumor cells. Comparative IVM analysis of these tumors directly shows the critical incorporation of the co-engrafted tissue into the stroma of the growing tumor and ultimately the pronounced importance of this stroma and unique microenvironment for tumor growth and angiogenesis.

## Methods

### Materials

All materials were obtained from Sigma-Aldrich (St. Louis, MO) unless otherwise noted.

### Animals

All animal experiments were performed in accordance with Institutional Animal Care and Use Commitee guidelines at Sydney Kimmel Cancer Center and Proteogenomics Research Institute for Systems Medicine. Athymic Nude- *Foxn1 ^nu^*, Balb/c, C57BL/6J and FVB mice from either Charles River Laboratories (Wilmington, MA) or Jackson Laboratories (Bar Harbor, ME) GFP C57BL/6J mice were a kind gift of Dr Christa Mueller-Seiburg (Burnham Institute). Tg(TIE2GFP)287Sato/J mice were purchased from Jackson Laboratories. Ten to fourteen week old female (80) and male (20) Athymic Nude- *Foxn1 ^nu^*, Balb/c (5), C57BL/6J (10), GFP C57BL/6J (5), Tg(TIE2GFP)287Sato/J (5) and FVB mice (20) were used for the dorsal skinfold implantations and donor tissues. Once the mice were ~25g, the chambers were placed on the dorsal skinfold and the mice were segregated into separate cages and monitored daily.

### Fluorescent tumor cell lines

All cell lines were grown at 37°C in 5% CO _2_ in air. N202 (gift from Joseph Lustgarten, Mayo Clinic, Scottsdale, AZ), MOVCAR-16 (gift from Denise Connolly, Fox Chase Cancer Center, Philadelphia, PA), TrampC2 (ATCC, Manassas, VA) and Lewis Lung Carcinoma (LLC - ATCC, Manassas, VA) cells were maintained in Dulbecco’s Modified Eagle Medium (DMEM) high glucose supplemented with l-glutamine (2 mM), penicillin (100 U/ml), streptomycin (100 U/ml), sodium pyruvate (1 mM) (Invitrogen, Carlsbad, CA) and 10% heat-inactivated Fetal Bovine Serum (Omega Scientific, Tarzana, CA). BT474 cells (ATCC, Manassas, VA) were maintained in Hybridoma-SFM supplemented with l-glutamine (2 mM), penicillin (100 U/ml), streptomycin (100 U/ml), sodium pyruvate (1 mM) (Invitrogen, Carlsbad, CA) and 10% heat-inactivated FBS (Omega Scientific, Tarzana, CA). The histone H2B-GFP was subcloned into the SalI/HpaI sites in the LXRN vector (Clontech, Palo Alto, CA) using SalI and blunted NotI sites from the BOSH2BGFPN1 vector ^21^. The monovalent cherry (mCherry) vector was created from the H2B-GFP vector by cloning the mCherry gene (Dr Roger Tsien, UCSD) to replace the green fluorescent protein (GFP) gene. GP2-293 cells were infected with vesicular stomatitis virus (VSV) and the H2B-GFP or H2B-mCherry-containing virus to produce viable virus. N202, BT474, TrampC2, MOVCAR-16 and LLC cells were transduced with the viable virus to stably incorporate the H2B-GFP or H2B-mCherry gene. The transduced cells were sorted twice using fluorescence-activated cell sorting (FACs) to ensure 100% of the cells stably expressed the H2B-GFP or H2B-mCherry protein.

### Tumor model

We used the classic IVM tumor model ^20^ with modifications. The mice, usually Athymic Nude- *Foxn1 ^nu^* mice (25–30 g body weight), were anesthetized (7.3 mg ketamine hydrochloride and 2.3 mg xylazine per 100 g body weight, intraperitoneal injection) and placed on a heating pad. As per the standard IVM tumor model ^20, 22^, a titanium frame was placed onto the dorsal skinfold of the mice to sandwich the extended double layer of skin. A 15 mm diameter full-thickness circular layer of skin was then excised. The superficial fascia on top of the remaining skin was carefully removed to expose the underlying muscle and subcutaneous tissue which was then covered with another titanium frame with a glass coverslip to form the window chamber. After a recovery period of 1–2 days, tumor spheroids were implanted.

Tumor spheroids were formed by plating 50,000 cells (N202, LLC, TrampC2 and MOVCAR-16) onto 1% agar-coated 96-well non-tissue culture treated flat bottom dishes (Becton Dickinson, Franklin Lakes, NJ) (20 μl cells in 100 μl medium) and centrifuging 4 times at 1200 g for 15 min, rotating the dish after every centrifugation. The cells were incubated an additional 3–7 days (depending on cell type) at 37°C in 5% CO _2_ in air to form tight 3-dimensional spheroids. BT474 cells required 500,000 cells in the presence of Matrigel (BD Bioscience, San Diego) (2:1 cell volume dilution - cells to matrigel) to form spheroids in culture.

The tumor spheroids were implanted in the window chamber directly onto the exposed dorsal skin either alone to created standard, classic, subcutaneous model or with lung, liver, mammary (lactating female mammary fat pad) or prostate tissue which was excised from a donor mouse and minced into small pieces in penicillin (10,000 U/ml) – streptomycin (10,000 μg/ml) solution. Unless noted otherwise, the co-implanted tissue was excised from donor mice syngeneic to the tumor cells used. One animal was usually enough to supply donor tissues for an experimental set of 15 animals except for the EO model in the case of prostate tissue when 3 animals were needed. Typically, the tumor spheroid was placed in the center of a bed of 1–2 mm of flattened minced tissue onto the subcutaneous tissue of each mouse. Tumors were allowed to re-vascularize over 7–14 days depending on model. For the BT474 cells, in some cases 10 μl of 5 mg/ml human 17β-estradiol (University of California, San Diego pharmacy) was injected subcutaneously twice weekly.

For adaptation to a new microenvironment, the tumors were allowed to re-vascularize as above. The tumor was removed and the fluorescent tumor cells were separated from non-tumor cells. New tumor spheroids were formed and re-implanted with donor mouse tissue as above. This was repeated two more times to reprogram the tumor to its new microenvironment.

### Intravital microscopy (IVM) and fluorescence confocal microscopy of tumors

After implantation, tumor spheroids were allowed to revascularize (12–14 days) and tumors were imaged with intravital fluorescence video microscopy, as described ^20^. The tumors were imaged with a FITC or Texas Red filter using an integrated frame grabber. Confocal microscopy was used to acquire dual fluorescence images via a Nikon E2000 microscope (20× and 60× objective lens) equipped with a Perkin Elmer UltraView 5ERS confocal system with an Hamamatsu Orca ER camera (Hamamatsu Corporation, Bridgewater, NJ). To construct movies, dual color images were taken every second; exposures for a single fluorophore were kept under 400 msec. Z-stacks were acquired every 0.5 µm and then resolved for 3D construction with Volocity LE v.3 software (Perkin Elmer).

### Tumor growth

Tumors were imaged using intravital fluorescence microscopy, as described as above. Tumor growth was analyzed off-line from the recorded, digital, grayscale 0-to-256 images using Image-Pro Plus (Media Cybernetics, Bethesda, MD). Tumor growth was determined in 2 ways, by measuring the area with fluorescence signal from the GFP or mCherry expressing tumor cells or by quantifying the cumulative fluorescence signal for the tumor over time. Tumor area is measured by counting the number of pixels with a grayscale intensity above 75, thereby making it easier to reliably follow irregularly shaped tumors. The cumulative tumor fluorescence signal was measured by signal summation of all pixels over 75. The tumor size is normalized to 1 based on the size of the tumor on day 1 after implantation. Even though the tumor spheroids are formed with approximately the same amount of tumor cells, one observes that during the course of their formation, the tumors were similar but not identical in size. Because we ultimately were interested in relative tumor growth over time between the different groups and experiments, we chose to simplify the growth curves by this standard normalization. In all cases, growth measured by area and aggregate fluorescence signal were found to be very similar.

### Mitotic and apoptotic indices

To determine mitotic and apoptotic indexes, two peripheral and two central X20 fields from 3 different animals for 6 random fields from the growing tumor in the dorsal skinfold chamber for each tumor/tissue combination was used. Only mitotic figures in metaphase-telophase (MI) are included in the mitotic index to exclude potential artifact of nuclear membrane distortion. Apoptotic/Pyknotic nuclei are defined as H2B-GFP labeled nuclei with a cross sectional area <30 μm ^2^. Nuclear karyorrhexis, easily distinguishable by the vesicular nuclear condensation and brightness of H2B-GFP, is included within this apoptotic index (AI). In the past we have compared our definition of apoptotic cells with tunnel assays and they were very similar in their assessment of apoptosis ^20^.

### Vascular parameters

To calculate the length and vascular density of tumors, photomicrographs obtained with the X10 objective were “flattened” to reduce the intensity variations in the background pixels and cropped to eliminate distorted areas. The blood vessels were morphologically obvious as dark channels between the fluorescent tumor cells. They were also identified by the presence of blood cells and circulating blood flow, which could easily be visualized in the movies from which the static images were made. During image processing, we adjusted the darkness thresholds to eliminate other nonfluorescent areas and highlight vessels exhibiting clear blood flow. The thresholding feature was used to segment the picture into objects and background. The picture was skeletonized in order to calculate vascular length. Vascular density was calculated as vascular length per tumor area.

### Statistics

SigmaStat (Systat Software, San Jose, CA) was used to determine statistical significance. Ranked ANOVAs with the Tukey post hoc test were used and a statistically significant difference delineated if p<0.05.

## Results and discussion

**Supplementary figure 1.**
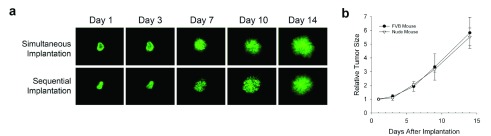
Effect of tissue implantation conditions on tumor growth. ( **a**) Fluorescence IVM images of simultaneous and sequential EO implantation of tumor spheroids. N202 tumor spheroids expressing H2B-GFP were implanted either simultaneously with the donor tissue or after the donor tissue was implanted and allowed to revascularize. Digital videos and static images were captured by IVM after implantation of the tumor spheroids on the days indicated. Tumor growth and vascularity were very similar between simultaneous and sequential implantations. ( **b**) Relative growth curves for EO tumors implanted in FVB versus nude mice. N202 tumor spheroids expressing H2B-GFP were co-implanted with FVB donor mammary tissue (syngeneic to tumor cell line) in either FVB or nude mice. Digital videos and static images were captured on the days indicated post tumor implantation. Tumor size was measured from total GFP fluorescence signal in each image to produce tumor growth curves relative to day 1. The growth of the tumors are very similar.

To develop new breast and lung tumor models that are amenable to continuous long-term, dynamic monitoring by IVM, yet maintain an “orthotopic” tumor microenvironment, we engrafted orthotopic tissue into the ectopic subcutaneous location, the dorsal skin with a window chamber already surgically attached, and then implanted tumor spheroids onto this donor tissue (see Methods). The tumor spheroids used in these EO tumors were formed from murine mammary adenocarcinoma (N202) and Lewis Lung Carcinoma (LLC) cells (see Methods). To follow tumor cell growth and chromosome dynamics independent from changes in the tumor stroma and surrounding host tissue, these tumor cell lines were transduced to stably express histone H2B linked to green (GFP) or mCherry fluorescent proteins. We observed very similar growth for the parental and stably transfected fluorescent tumor cells and when tumor spheroids were implanted simultaneously with engrafted tissue or onto engrafted tissue that had already revascularized days earlier ( Supplementary Figure 1a). We also assessed if the tumors implanted in syngeneic mice had a growth advantage over tumors implanted in nude mice. When we implanted syngeneic tumor cell spheroids in non-immunocompromised versus nude mice, we did not observe any noticeable differences in EO tumor growth ( Supplementary Figure 1b). Both the N202 and LLC cells grew very similarly in nude mice as in FVB and C57BL/6J mice, respectively ( Supplementary Figure 1b and data not shown). Consistent with this result, classic subcutaneous IVM tumor models routinely use nude mice in part because of several key advantages: i) enables implantation of a wide variety of tumor spheroids and tissues of different strains and species and ii) their abundant and hairless skin makes it easier to implant the titanium window chambers and observe the progressing tumors. Therefore, for the remaining experiments, we used fluorescent tumor spheroids implanted concurrently with other tissues in nude mice.

**Figure 1.**
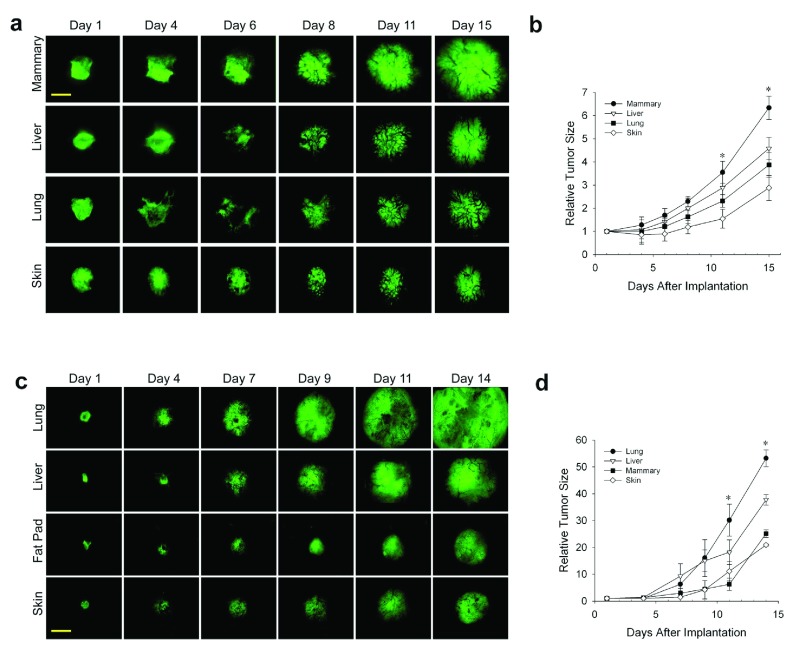
Effect of tissue microenvironment on tumor growth and development. ( **a**– **d**) N202 ( **a** and **b**) and LLC ( **c** and **d**) tumor spheroids expressing H2B-GFP were implanted directly onto the dorsal skin or with mammary, liver or lung tissue as indicated and monitored through the dorsal skin window chamber by IVM at the indicated times. ( **b** and **d**) Relative tumor growth curves. Tumor size on the day indicated was calculated by measuring the total GFP fluorescence signal in each image and dividing this signal by the GFP signal detected one day after implantation (day 1) (see Methods). Scale bar = 500 μm. Mean +/- SD are shown. * = p<0.05. n = 3–4 mice for all experiments.

In the last decade or so, it has become clear that the stroma and tissue microenvironment can affect tumor development. Our ability to co-implant other tissues from normal organs with different tumor cell types in the dorsal skin window chamber provides a unique way to study the direct effects of different tissue stromas on tumor development. Moreover, this system facilitates direct imaging of the tumors over many days using IVM. To assess the effects of different tissues on the tumor growth *in vivo*, tumor spheroids were implanted directly onto the dorsal skin alone in the window chamber (as per the classic IVM subcutaneous tumor model ^22^) or with different donor tissue. IVM enabled detailed visualization and quantification of tumor cell fluorescence signal as well as tumor area to assess tumor growth (see Methods). The N202 spheroids grew well on skin alone, better with each co-implanted donor tissue and most robustly with the orthotopic mammary fat pad tissue ( Figure 1a and b). After 15 days, tumors grown in mammary tissue were >3 times the size of tumors grown subcutaneously. Thus, the orthotopic tissue provided the heartiest environment for tumor growth.

Supplementary Movies S1 and S2. Distinct imaging of tumor cells, tissue stroma and vasculature. H2B-mCherry N202 mammary tumor spheroids (red) were implanted on mammary tissue from a GFP mouse (green). 20 days post-implantation, 3D confocal fluorescent microscopy images were constructed of the tumor and tissue stroma as described in the methods.

Having shown previously that prostate tumor cells grow better when implanted with prostate tissue that express key hormones required for tumor cell growth ^20^, we were concerned that robust growth could similarly emanate from hormones expressed in the mammary fat pad tissue. To avoid typical hormonal effects and to show that the enhanced tumor growth with co-engrafted orthotopic tissue was not restricted to one cell type, we created a new lung tumor model by implanting LLC tumor spheroids onto skin alone or co-engrafted with lung, liver, and mammary tissue ( Figure 1c). Fluorescent IVM again showed the tumors growing sooner and more rapidly with orthotopic tissue. At 14 days after implantation, tumors with orthotopic tissue were again at least three times larger than subcutaneous tumors ( Figure 1d). However, it should be noted for both LLC and N202 cells that after about a 10-day lag period, the growth rate of the subcutaneous tumors increased dramatically to become more similar to that of the EO tumors.

**Figure 2.**
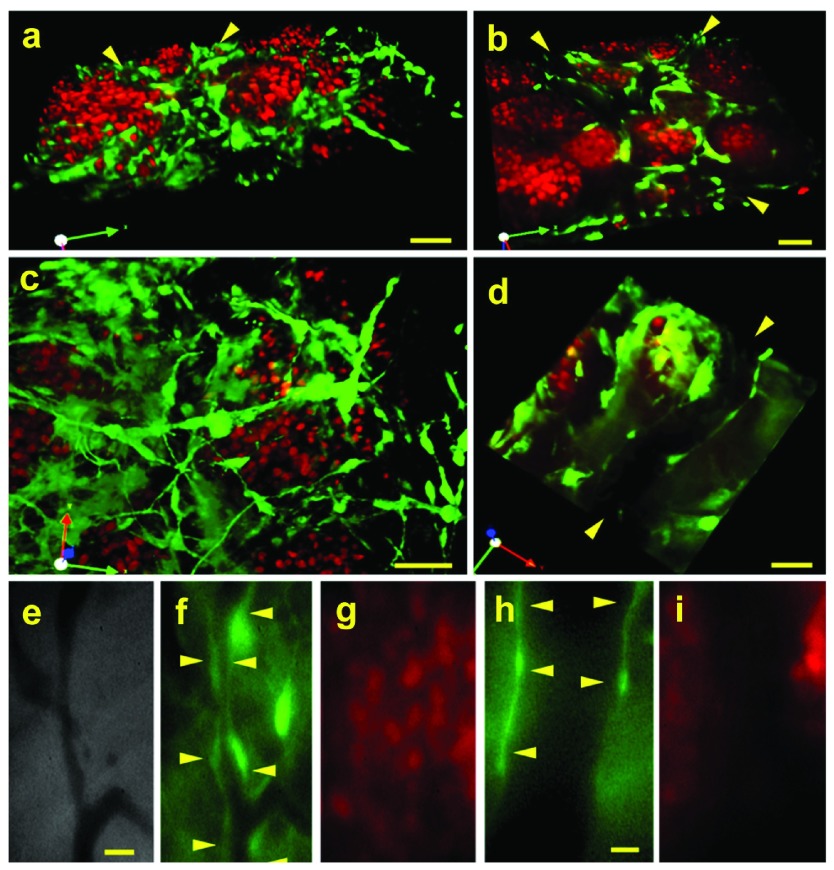
Distinct fluorescence imaging of tumor cells, tissue stroma and vasculature. N202 H2B-mCherry mammary tumor spheroids (red) were implanted with mammary tissue excised from a lactating GFP mouse (green) or from a mouse expressing GFP under the EC-specific TEK promoter (TEK-GFP). 20 days post-implantation, 3D confocal fluorescent microscopic images were constructed ( **a**– **d**: GFP, **h** and **i**: TEK-GFP) as well as direct IVM fluorescent microscopic images ( **e**– **g**: GFP) of the tumor and tissue stroma. Blood vessels are indicated by arrowheads. Scale bars = 10 μm ( **a**– **d**), 20 μm ( **e**– **g**), 5 μm ( **h** and **i**).

The EO tumor model described here uses three sources of tissue, the engrafted orthotopic tissue from the donor mice, the tumor spheroids implanted onto the engrafted tissue, and the entire living host mouse. To visualize the implanted tissue cells distinctly from the neoplastic tumor cells and to determine which tissue (engrafted tissue or host tissue) gives rise to the stroma and vasculature inside the EO tumors, donor mammary tissue from GFP transgenic mice ^23^ was implanted simultaneously with N202 tumor spheroids expressing H2B-mCherry. We explicitly used donor tissue from GFP-mice so that we could definitively detect, via fluorescence imaging, which parts of the tumor stroma its cells and vessels were from (i.e. the implanted tissue versus the host mouse). Confocal fluorescence microscopy showed very typical solid tumor architecture with well-separated islands of “red” tumor cells surrounded by “green” stromal cells. When the images were projected in 3D, green vasculature with other cells derived from the orthotopic stroma could also be seen weaving amid the red tumor cells ( Figure 2a–d; Supplementary Movies 1 and 2). Vascular tubes with blood flow were also readily apparent in phase images as dark vessels against the lighter stroma ( Figure 2e). Under fluorescent microscopy, the blood vessels within tumors were uniformly lined with cells expressing GFP ( Figure 2a, b, d and f) and were clearly distinct from tumor cells expressing mCherry ( Figure 2a, b, c and g). Thus, the engrafted tissue persists to become the supportive stroma for the tumor cells in this EO model. Furthermore, these images show not only that a thriving tumor has been created with very typical, quite classic morphology but that two key components of the tumor can be marked *a priori* to be visualized distinctly in a long-term, dynamic, continuous imaging system.

Effect of tissue microenvironment on tumor growth and development. N202 and LLC tumor spheroids expressing H2B-GFP were implanted directly onto the dorsal skin or with mammary, liver or lung tissue as indicated and monitored through the dorsal skin window chamber by IVM at the indicated times.

To examine the vascular endothelium more specifically, we also implanted donor tissue excised from mice expressing GFP under the endothelial cell-specific promoter TEK ^24^. Here again, the tumor vasculature was clearly lined with GFP-expressing endothelial cells ( Figure 2h) that were clearly distinct from tumor cells ( Figure 2i). The green vessels attached to host vessels lacking GFP and blood cells circulated seamlessly between the contiguous vessels. The tumor stroma and neovasculature, therefore, arose from the engrafted donor tissue and successfully revascularized by attaching to the unlabeled vessels present in the host animal.

**Figure 3.**
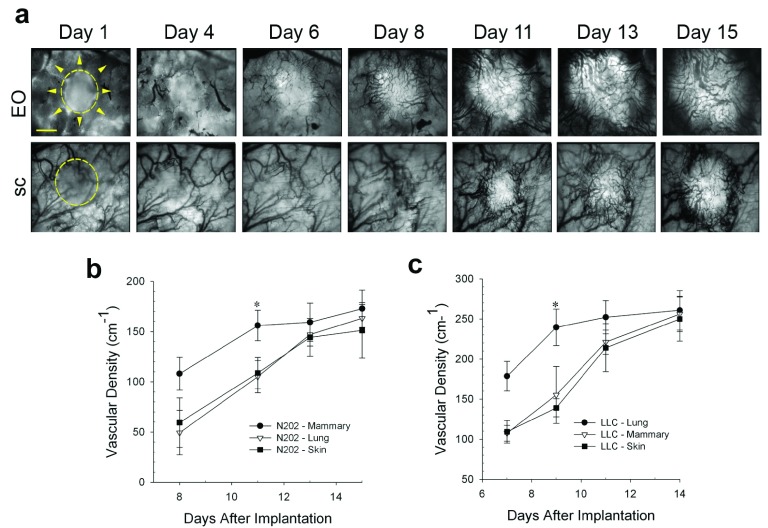
Effect of tissue co-engraftment on tumor vascular development. ( **a**) Phase images of N202 tumor spheroids expressing H2B-GFP that were implanted directly onto the dorsal skin (sc) or with donor mammary tissue (EO) as indicated and monitored by IVM at the indicate times after implantation. N202 ( **b**) and LLC ( **c**) tumor vascular densities were measured at the indicated times (see Methods). Scale bar = 10 μm. Mean +/- SD are shown. * = p<0.05. n = 3–4 mice for all experiments.

Tumor growth ultimately requires vascular development to fulfill the metabolic demands of the cancer cells ^25^. To compare the rates of revascularization, tumors growing in different tissue microenvironments were transilluminated so that dark blood vessels were readily visible against the bright tumor background ( Figure 3a). The vascular development of N202 tumors grown either subcutaneously or on implanted lung tissue lagged for days behind the N202 tumors grown on orthotopic mammary tissue. Eventually, the vascular density became nearly equivalent by about 2 weeks in both models ( Figure 3a and b). Blood vessels developed similarly for the LLC tumors ( Figure 3c). Vascularization occurred sooner and initially was more rapid and extensive in EO tumors, likely supporting more rapid tumor growth.

Effect of tissue co-engraftment on tumor vascular development. N202 and LLC tumor vascular densities measured at the indicated times.

**Supplementary figure 2.**
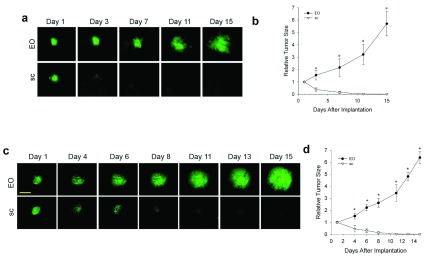
Effect of orthotopic tissue co-implantation on TRAMP C2 and MOVCAR16 tumor growth. Fluorescence images ( **a** and **c**) and growth curves ( **b** and **d**) of TRAMPC2 ( **a** and **b**) and MOVCAR-16 ( **c** and **d**) tumor spheroids expressing H2B-GFP that were implanted directly onto the dorsal skin (sc) or with respective orthotopic donor tissue (EO) (prostate and ovary respectively) and monitored through the dorsal skin window chamber by IVM at the indicated times. ( **b** and **d**) Relative tumor fluorescence signal was measured by total GFP intensity relative to day 1. Scale bar = 500 μm. n = 3–4 mice for all experiments.

We also tested other tumor cell lines in this system to create prostate and ovarian EO tumor models and they showed quite extreme behavior with an extraordinary dependence on co-implantation of the correct orthotopic tissue. Both Tramp-C2 prostate tumor cells ( Supplementary Figure 2a) and MOVCAR-16 ovarian tumor cells ( Supplementary Figure 2b) did not grow at all when implanted alone subcutaneously in the dorsal skin window chambers. They actually disappeared over a 10-day period. However, when co-implanted with their proper orthotopic tissue in the EO model, they both grew very well, with vascular development clearly evident by 6 days after implantation. Co-implantation with other ectopic tissues did not prevent tumor disappearance (data not shown). Thus, it is not just the presence of any co-implanted stroma tissue to envelop the tumor spheroid that is necessary for growth. These tumor cell lines appear actually to require the co-engraftment specifically of the orthotopic tissue to grow and to develop new blood vessels in the tumor. The orthotopic tissue co-implantation can essentially rescue *in vivo* growth and enable tumor cell lines to create a more robust and potentially useful tumor model *in vivo*.

**Figure 4.**
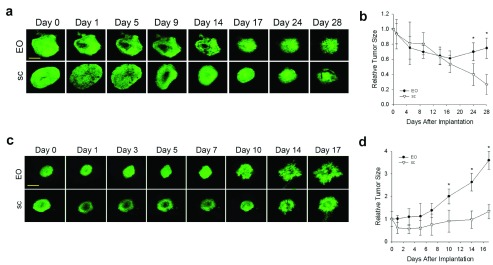
Effect of mouse orthotopic tissue co-engraftment on human BT474 tumor growth. BT474 tumor spheroids expressing H2B-GFP were implanted directly onto the dorsal skin (sc) ( **a** and **b**) or with mouse mammary fat pad donor tissue (EO) ( **c** and **d**) and monitored by IVM. ( **a** and **c**) Fluorescence IVM images on the indicated days after implantation. ( **b** and **d**) Relative tumor growth curves. Tumor size was measured on indicated days based on total GFP fluorescence signal relative to day 1 as in Figure 1. Mice received human estrogen supplement in ( **c** and **d**) but not ( **a** and **b**). Scale bar = 500 μm. n = 3–4 mice for all experiments.

We also observed that human tumor cells can also exhibit a strong preference for orthotopic tissue co-implantation. We implanted the well-known human breast cancer cell line BT474 as tumor spheroids in the dorsal skin window chamber with and without mouse mammary tissue. First, we did so without supplementing the mice with human estrogen, which is customary for these tumor cells. Figure 4a and b show that the tumors did not grow well and substantially regressed from the original tumor spheroid, especially in the subcutaneous-only implants. However, with mammary tissue, the tumor regression was reversed after 2 weeks with modest growth thereafter. When we performed the implantations this time with estrogen supplementation, tumor growth was much more robust. Figure 4c and d show that, again, the fluorescent tumors grew more quickly in the EO model than subcutaneous model. The tumor spheroids decreased in size initially in the subcutaneous model for about 1 week and then grew modestly thereafter. The EO tumors did not regress and required about 5–7 days to begin robust growth. Angiogenesis was readily evident by 8–10 days after implantation. Vascular development lagged along with little tumor growth in the subcutaneous tumors alone until after 2 weeks. Thus, it appears that human tumor cells can also benefit from orthotopic mouse tissue implantation quite similarly to the mouse tumor cell lines. Even without human estrogen supplementation, these tumors cells did better in the orthotopic stroma milieu ( Figure 4a and b). Then, also with human estrogen, the tumors grew much better when exposed to an orthotopic tissue environment. Our new IVM study of multiple tumor types subjected to tissue co-implantation clearly shows that the tissue stroma can have a very significant and even dramatic effect on tumor growth and vascular development. Ultimately, every tumor type tested grew best when co-implanted with respective orthotopic tissue.

Effect of mouse orthotopic tissue co-engraftment on human BT474 tumor growth. BT474 tumor spheroids expressing H2B-GFP were implanted directly onto the dorsal skin (sc) or with mouse mammary fat pad donor tissue (EO) and monitored by IVM. Tumor growth was measured by fluorescence signal over time. Mice received human estrogen supplement.

**Figure 5.**
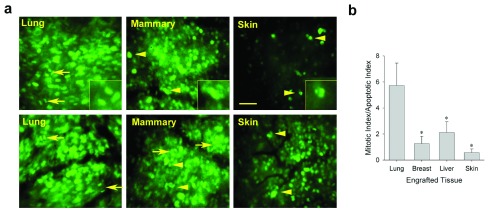
Effect of tissue co-engraftment on mitotic/apoptotic indices of tumor cells. ( **a**) Higher magnification fluorescence micrographs showing LLC tumor cells growing in indicated tissues (9 days post-implantation) to assess their effect on relative tumor cell mitosis and apoptosis. Mitotic (arrows) and apoptotic (arrowheads) cells were counted to determine the ratio of mitotic cells to apoptotic cells ( **b**). Scale bar = 100 μm. Mean +/- SD are shown. * = p<0.05. n = 3–4 mice for all experiments.

Using IVM with the H2BGFP-labeled tumor cells allowed us to visualize directly the growing tumor cells and their fluorescent nuclei in real time. To begin to examine the cellular mechanisms mediating growth differences in the distinct tissue microenvironments, chromatin dynamics were imaged to quantify both mitotic and apoptotic cells in the LLC spheroids implanted with lung tissue, ectopically with other tissues, or subcutaneously, directly on skin ( Figure 5a). The ratio of mitotic to apoptotic tumor cells in each tumor revealed that the LLC tumors growing on orthotopic tissue had a strong bias towards mitosis ( Figure 5b). LLC tumors growing in mammary tissue, liver or skin had a more balanced ratio of mitosis to apoptosis. The N202 tumors showed very similar results whereas the TRAMP-C2 and MOVCAR-16 tumors also exhibited ample mitosis in the EO model, but no mitosis and ample apoptosis and cell death, as they disappeared when implanted alone subcutaneously ( Supplementary Figure 2). Thus, the orthotopic tissue could create for multiple tumor cell lines a local tissue microenvironment that favored tumor growth by promoting tumor-cell mitosis over apoptosis.

Effect of tissue co-engraftment on mitotic/apoptotic indices of tumor cells. Measurements of mitotic and apoptotic indicies in tissue from lung, breast, liver and skin.

**Figure 6.**
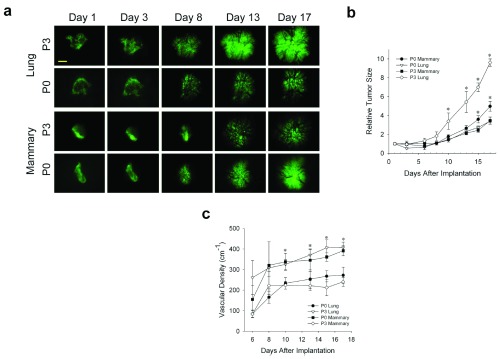
Tumor cell adaptation to non-orthotopic tumor microenvironment. ( **a**) N202 tumor spheroids were passaged 0 or 3 times on lung tissue, as described in methods, and implanted onto engrafted lung or fat pad tissue and monitored by IVM at the indicated times. ( **b**) Relative tumor growth curves. Tumor size was determined by measuring tumor area from fluorescence images on the indicated days and normalizing this relative to day 1 (see Methods). ( **c**) Tumor vascular density of the indicated passaged N202 tumor spheroid was also determined as described in the methods. Scale bar = 500 μm. Mean +/- SD are shown. * = p<0.05. n = 3–4 mice for all experiments.

In humans, tumors are not restricted to one organ, but instead eventually reprogram to alter their phenotype often in order to metastasize to other organs. This well-known characteristic of cancer suggests tumor cells have the inherent ability to genetically adapt and maybe even to grow optimally in other non-orthotopic tissue environments. To determine the ability of tumor spheroids to adapt to different tissue microenvironments, we passaged N202 mammary tumor spheroids on donor lung tissue in the dorsal window chamber model (as described in the methods). Initially, mammary tumors grew poorly ( Figure 6a and b) and revascularized more slowly ( Figure 6c) when grown on lung tissue than orthotopic mammary tissue. However, after three passages of growing in lung tissue implanted in the IVM chamber followed by isolating and re-culturing the tumor cells for spheroid formation and then re-implantation, mammary tumor cells eventually grew much more robustly and revascularized faster ( Figure 6a–c) on donor lung tissue. Remarkably, when lung-adapted mammary tumor cells were implanted onto mammary tissue, the tumors grew quite poorly and revascularized rather slowly ( Figure 6a–c). In fact, their growth and revascularization was similar to the growth on the lung tissue prior to being trained via lung tissue passaging. Thus, interactions between tumor cells and stroma become evident, including tumor cells adapting to a new tissue microenvironment and eventually reaching a new phenotype optimized for the new stroma, but no longer flourishing in the original orthotopic tissue.

Tumor cell adaptation to non-orthotopic tumor microenvironment. Tumor size was measured by total tumor area relative to day 1. Tumor vascular density of the passaged N202 tumor spheroid was also determined.

IVM offers an unparalleled view into tumor development, allowing dynamic, high resolution, *in vivo* imaging of molecular and cellular events. Here, we greatly expand the relevancy of the classic IVM tumor model by introducing orthotopic tissue into the dorsal skinfold chambers, thereby creating EO tumor models allowing easy and direct manipulation of the tissue microenvironment that can now be viewed with a long-term, dynamic, continuous imaging system. We show that tumors in an orthotopic tissue microenvironment grow more robustly and develop vasculature more rapidly than subcutaneous and other ectopic tissue models. The orthotopic environment facilitates tumor cell mitosis over apoptosis. As new blood vessels are needed to support tumor growth, the faster growing blood supply observed in the EO models likely supports the greater rates of mitosis in the tumor cells growing with orthotopic tissue versus just subcutaneously. One way to think of why these tumors develop differently is that in classic subcutaneous tumors the implanted tumor spheroid communicates with the surrounding tissue to induce accommodations that are required for tumor growth, including angiogenesis. As it is the only supplicant in this case, perhaps the tumor cannot fully facilitate a normal tissue wound-repairing process by itself. In co-implantation, the donor tissue not only communicates to the surrounding tissue in a similar way to the tumor spheroid, but also possesses important elements, including key cells and blood cells, that could prime the tissue. As a minced, wounded tissue in need of repair, it appears to be able to revascularize, in part through anastomosis of its vessels, with underlying blood vessels of the skin. This donor tissue can do so on its own as we reported previously ^19^ and with the tumor spheroid where the two appear to work quite well together to create a functioning robust neoplastic tissue. When comparing tumors with and without the donor orthotopic tissue, it appears clear that the co-implanted stroma helps the tumor take root more quickly with faster development of functioning blood vessels leading to a significant growth advantage, at least initially. It will be interesting to see how similar or not the EO and subcutaneous tumors are over time; once the subcutaneous tumors have overcome their longer lag period and achieve similar vascular densities and growth, does the incorporated orthotopic stroma contribute to sustained, long term, meaningful differences between the two models?

Recreating the orthotopic tumor microenvironment in the dorsal skinfold window chamber is a significant advancement that maintains the power of the IVM imaging system. This approach incorporates the more relevant orthotopic tissue microenvironment, while still being amenable to dynamic imaging by IVM. The IVM experiments show possible improvements over subcutaneous tumor models and provide key direct evidence that the tumor stroma and microenvironment can dramatically influence growth and angiogenesis.

Though tumor models abound, one of the many strengths of this novel EO model is its ease of use. True orthotopic tumor models, in which tumors are implanted onto orthotopic tissue, can be technically difficult to create. For example, it is quite challenging to inject mammary tumor cells into the very tiny mammary tissue of the mouse, especially when the cell number or injection volume is similar to that of mouse tissue. Genetic tumor models are complicated and costly to create and are specialized for a very specific set of genetic defects. Dynamic *in vivo* imaging, especially at the cellular level, is also very limited in many of these models. The EO model overcomes each of these difficulties. Engrafting tissue in the dorsal skinfold chamber is fairly straightforward. Numerous types of tumors and donor orthotopic tissue can be readily implanted. This model is widely applicable to many tumor types and is amenable to dynamic imaging by IVM, which offers an unparalleled view into tumor development, allowing dynamic, high resolution, *in vivo* imaging of molecular and cellular events.

Importantly, recreating the orthotopic tumor microenvironment in the dorsal skinfold window chamber allows researchers to focus on tumor-stroma interactions in a more controlled environment. Growing tumor spheroids in different microenvironments revealed that tumors in an orthotopic tissue microenvironment grow more robustly than subcutaneous and other ectopic tissue models. The orthotopic environment facilitates tumor cell mitosis over apoptosis. As new blood vessels are needed to support tumor growth, this faster growing blood supply likely supports greater rates of tumor-cell mitosis in tumor cells growing orthotopically versus subcutaneously. However, growth in a single microenvironment is not hardwired into the tumor cell. Tumor cells clearly have the ability to adapt to new microenvironments. Thus, these new tumor models may allow the ongoing interaction between tumor and stroma to be examined in greater detail and with more precise control than previously possible. Further experimentation comparing EO versus subcutaneous tumors may be warranted. Such studies may find additional functional and molecular distinctions that not only uncover stroma effects, but also provide contrasts to actual tumors in humans.

## Supplementary materials

Effect of tissue co-engraftment on tumor development. Comparison of the relative tumor size of ectopic/orthotopic implantation of tumor spheroids in FVB versus nude mice. N202 tumor spheroids expressing H2B-GFP were implanted in FVB or nude mice with the donor mammary fat pad tissue, allowed to revascularize, and imaged by IVM to measure tumor size by total fluorescence signal on the indicated day post tumor implantation. Again, the growth of the tumors are observed to be very similar.

Effect of orthotopic tissue co-implantation on TRAMP C2 and MOVCAR16 tumor growth. Growth curves of TRAMPC2 and MOVCAR-16 tumor spheroids expressing H2B-GFP that were implanted directly onto the dorsal skin (sc) or with respective orthotopic donor tissue (EO) (prostate and ovary respectively) and monitored through the dorsal skin window chamber by IVM at the indicated times.
