# Supplementary material for: Co-implanting orthotopic tissue creates stroma microenvironment enhancing growth and angiogenesis of multiple tumors
Source: F1000Res. 2013 Aug 29;2:129. Originally published 2013 May 21. [Version 2] doi: 10.12688/f1000research.2-129.v2 (PMC3976108; doi:10.12688/f1000research.2-129.v2)
Supplement: Peer review history

# Peer review: Referee response for version 2

**Published:** 2014-03-05

**Authors:** Hong Zhao

**Affiliations:** 1 Department of Systems Medicine and Bioengineering, Methodist Hospital Research Institute, Houston, TX, USA

I have read this submission. I believe that I have an appropriate level of expertise to confirm that it is of an acceptable scientific standard.

# Peer review: Referee response for version 2

**Published:** 2014-01-23

**Authors:** Erik Sahai

**Affiliations:** 1 Tumour Cell Biology Laboratory, Cancer Research UK, London, UK

The authors have responded well to my comments; I approve this version also.

I have read this submission. I believe that I have an appropriate level of expertise to confirm that it is of an acceptable scientific standard.

# Peer review: Referee response for version 1

**Published:** 2013-06-27

**Authors:** Erik Sahai, Danielle Park

**Affiliations:** 1 Tumour Cell Biology Laboratory, Cancer Research UK, London, UK; 2 Cancer Research UK, London, UK

This study expands on long-standing observations that tumor xenografts behave differently in different anatomical locations. The hypothesis is that there are some paracrine interactions that favour tumorigenecity that can only occur with cells or matrix from the appropriate tissue location. Many studies have suggested this over the past twenty five years, for example Nakamura *et al.* (2007), Killion *et al.* (1999), Fidler et al. (1990). This study revisits the theory in a methodical manner. Tumor cell lines from different anatomical locations are grafted with or without normal tissue from the same organ. Lung, ovarian and breast cancer cells are found to grow quicker when co-implanted with normal tissue from the lung and breast, respectively. In general, the work is thorough and convincing, although there is some scope for improvement and clarification.

Technical comments

1. Borgstrom *et al.*, use area of fluorescence signal and/or cumulative fluorescence signal as a measure of tumour size and growth (Figures 1a-d, 4a-d, and 6 a-b and Supplementary 1 a-b and 2a-d). Fluorescent signal will be attenuated at increasing depths, particularly when using low wavelength/low penetrance fluorophores such as GFP. This method is then inappropriate for irregularly shaped tumours that grow into the body of the mouse rather than just expanding along the plane of the window. In such instances tumour size will be underestimated. Ex-vivo analysis of tumour size/weight may have been more accurate.

2. Borgstrom *et al.*, define apoptotic cells by the size of the nucleus (upper cut off of <30µm ^2^). Is the same threshold used for all cells lines? If so, is this appropriate? When measuring karyorrhexis is each nuclear fragment counted as one apoptotic cell? As tumour cells lack a cell membrane marker, it is impossible to know whether several nuclear fragments arose from a single apoptotic cell, or several. This would have been better supported using a fluorescent probe for phosphatidyl serine, activated caspase or loss of plasma membrane integrity. 

3. Borgstrom *et al.*, indicate that ‘green vessels (from donor orthotopic tissue) attached to host vessels lacking GFP and blood cells circulated seamless between the contiguous vessels’- 2i: this claim is not well supported by figure. The use of shadows as a measure of vascular density is also not ideal, it would have been better demonstrated by the injection of fluorescent vascular tracers in addition also ex vivo staining of thick sections with endothelial cell markers. For comparison, another study has recently reported imaging of endothelial cells in syngeneic tumor grafts: http://www.landesbioscience.com/journals/intravital/article/24790/?show_full_text=true .

We have read this submission. We believe that we have an appropriate level of expertise to confirm that it is of an acceptable scientific standard.

# Response

**Published:** 2013-08-08

**Authors:** Phil Oh

**Affiliations:** Proteogenomics Research Institute for Systems Medicine, USA

We would like to thank the reviewers for their time and comments.

1. We agree that tissue attenuation of fluorescence signal is likely and could contribute to an underestimation of tumor growth. But that is why we performed two different measurements, including one based on tumor area which is defined by the pixels reaching a minimum threshold fluorescence signal from the glowing tumor cells and thus will be much less sensitive to such attenuation. The results were very similar with both measurements. Consistent with this, these tumors tend to grow more in 2-dimensions and be a bit flattened and not so spherical in shape, in part because of the glass coverslip. Not sure that this issue is all that critical to our findings and conclusion; for the purposes of comparisons made in the paper, we used the same methods which quantified growth differences that were not particularly subtle but rather quite obvious from the captured images. Performing ex- *vivo* measurement of tumor size and weight somewhat defeats the overall purpose behind using IVM and this model system. We wish to get dynamic and continuous intravital data on the tumors at multiple scales and ultimately avoid using huge numbers of animals to get data that may only be a bit more accurate but at such a considerable cost in many different ways. There are some advantages here: because we are using tumor cells that provide the fluorescent signal specifically, we can be sure that our measurement reflects actual tumor cells and not other events that can appear to contribute to tumor size and apparent growth such as dead cells, infiltration of other cells, hemorrhage, edema, etc. Here we wish to examine effects of stroma/tissue implantation on the tumor cells themselves in- *vivo* and their proliferation. So our approach is more direct and maybe even better in many respects than simple tumor excision and weighing.  
2. We have compared our definition of apoptotic cells with tunnel assays and they were very similar in their assessment of apoptosis. This was reported in reference 20. We have added a sentence in this regard in the methods citing this paper. 
3. We are able to observe the blood including cells circulating through unmistakable blood vessels. The static images shown were taken from our movies. When we set up these measurements, we picked darkness thresholds that highlighted unambiguous vessels with clear blood flow. We have frequently used various fluorescent tracers which as expected provide a signal that coincides with the blood flow seen through the vessels. But this extra procedure on the mice ultimately was unwarranted for this singular purpose because it did not really augment or refine our measurement of vascularity. Again ex vivo evaluations seem contrary to noninvasive, dynamic, continuous, in vivo imaging attained here and would likely add more effort and animals but little beyond the results and conclusions provided more efficiently through IVM. We appreciate these comments and have added further description of the vascularity measurement in the methods to provide more clarity to the reader.

# Peer review: Referee response for version 1

**Published:** 2013-06-14

**Authors:** Hong Zhao, Zhen Zhao

**Affiliations:** 1 Department of Systems Medicine and Bioengineering, Methodist Hospital Research Institute, Houston, TX, USA; 2 The Methodist Hospital Research Institute, Houston, TX, USA

The article provides a novel way to image and investigate the tumor in the original similar microenvironment. That makes it much easier to investigate the tumor characteristics related to the microenvironments. 

However, there are some questions need to be clarified in the paper:

1. After the implantation of the orthotopic tissue to the dorsal skin chamber, where is the blood supply of the tissue from? Will the angiogenesis happen in the tissue like a tumor? Usually, it’s a challenge to make the engrafted normal tissue to get good blood supply after the implantation. If the angiogenesis or vasculogenesis happened in the tissue, where did the vessels arise from? Were they from the host animal or just attached to the blood vessel of the host animal?

2. For the vascular parameters, I didn’t notice any vascular mark methods, were they only identified by the dark space between the fluorescent tumor background? If so, will the stroma tissue in the tumor which has no fluorescent effect the counting of the blood vessels?

3. There are no units of the tumor size in the graphs of figure 1, 4, 6 and the supplement figures.

We have read this submission. We believe that we have an appropriate level of expertise to confirm that it is of an acceptable scientific standard, however we have significant reservations, as outlined above.

# Response

**Published:** 2013-08-08

**Authors:** Phil Oh

**Affiliations:** Proteogenomics Research Institute for Systems Medicine, USA

We appreciate these comments and have revised the text description to be clearer. We have also added a paragraph to the discussion that describes possible process differences between EO versus subcutaneous models.

1. We explicitly use donor tissue from GFP-mice so that we can definitively detect via fluorescence imaging, which parts of the tumor stroma its cells and vessels are from, the implanted tissue versus the host mouse. Moreover we also utilize tumor cells that express mCherry (red signal) for further distinction. It is clear from the figure 2 that there is ample donor tissue represented in the stroma of these tumors. Then when we look specifically at blood vessels which are unambiguously identified by their distinct structure and the flow of blood cells through them, we readily observed that the endothelial cells lining these blood vessels fluorescence “green” from their GFP expression. To increase certainty that the cells of the vascular wall in the tumor were indeed from the implanted donor orthotopic tissue, we performed additional experiments where the tissue was donated once again from transgenic mice expressing GFP but this time the expression was more selective to cell type because it is controlled by an endothelial cell promoter. Again the vessel wall cells expressed the GFP in the tumors. Please also see manuscript text for fig. 2 which describes this directly. For example we state: “To examine the vascular endothelium more specifically, we also implanted donor tissue excised from mice expressing GFP under the endothelial cell-specific promoter TEK24. Here again, the tumor vasculature was clearly lined with GFP-expressing endothelial cells (Figure 2h) that were clearly distinct from tumor cells (Figure 2i). The green vessels attached to host vessels lacking GFP and blood cells circulated seamlessly between the contiguous vessels. The tumor stroma and neovasculature, therefore, arose from the engrafted donor tissue and successfully revascularized by attaching to the unlabeled vessels present in the host animal.” One way to think of this process is that in classic subcutaneous tumors the implanted tumor spheroid is communicating with the surrounding tissue to induce accommodations that are required for tumor growth and that include angiogenesis. It is the only supplicant for proper adaptations and maybe lacks all the means possible in a normal tissue wound repairing process. With co-implantation, the donor tissue not only communicates its similar needs but also inherently has important elements, including key cells and blood vessels, to provide in a primed state to form the surviving tissue. As a minced, wounded tissue in need of repair, it appears to be able to revascularize in part through anastomosis of its vessels with underlying blood vessels of the skin. This donor tissue can do so on its own as shown in our 2007 Nature Biotechnology article in which we state that the donor implanted tissue’s blood vessels “maintained both tissue- and species-specificity, even expressing key organ-specific biomarkers”. This donor tissue can also do so with the tumor spheroid where the two appear to work quite well together to create a functioning robust neoplastic tissue. When comparing tumors with and without the donor orthotopic tissue, it appears clear that the co-implanted stroma helps the tumor take root more quickly with faster development of functioning blood vessels leading to a significant growth advantage at least initially. It will be interesting to see how similar or not the EO and subcutaneous tumors are over time; once the subcutaneous tumors have overcome their longer lag period and achieve similar vascular densities and growth, does the incorporated orthotopic stroma contribute to sustained, long term meaningful differences between the two models? 
2. We can readily differentiate the blood vessels from the non-fluorescent stroma because the blood vessels not only encompass morphologically distinct and obvious dark channels between the fluorescent tumor cells but also were identified by presence of blood cells and even actual circulating blood flow, which could easily be visualized in the movies from which the static images were made. For our measurement, we picked darkness thresholds that emphasized vessels with clear blood flow. As expected, when we have used various fluorescent tracers they provided a signal that readily coincided with the blood flow seen through the blood vessels. We appreciate these comments and have attempted to be clearer by adding more details on this to the methods section.  
3. The tumor size is based on the size of the tumor on Day 1 after implantation which is normalized for each tumor to be 1. Therefore, the relative tumor growth graphs do not have any units in the Y-axis. You can see from the pictures provided that the tumors were similar but not identical in size (in part because the tumor spheroids,at the time of implantation, cannot be matched perfectly in size). Because we ultimately were interested in relative tumor growth over time between the different groups and experiments, we choose to simplify the growth curves akin to many other past published studies by this standard normalization. How we measure tumor size and growth including this normalization is described in the methods section with a brief sentence in some of the figure legends. Please note that we measured size in two ways and both gave very similar results. We appreciate these comments and have adjusted the legends and methods to be clearer.
